# 3Y-TZP/Ta Biocermet as a Dental Material: An Analysis of the In Vitro Adherence of Streptococcus Oralis Biofilm and an In Vivo Pilot Study in Dogs

**DOI:** 10.3390/antibiotics13020175

**Published:** 2024-02-09

**Authors:** Anton Smirnov, Oleg Yanushevich, Natella Krikheli, Nestor Washington Solis Pinargote, Pavel Peretyagin, Sergey Grigoriev, Luis Alou, David Sevillano, Roberto López-Piriz, Francisco Guitian, José Florindo Bartolomé

**Affiliations:** 1 Spark Plasma Sintering Research Laboratory, Moscow State University of Technology “STANKIN”, Vadkovsky per. 1, Moscow 127055, Russia; nw.solis@stankin.ru (N.W.S.P.); p.peretyagin@stankin.ru (P.P.); s.grigoriev@stankin.ru (S.G.); 2 Scientific Department, A.I. Evdokimov Moscow State University of Medicine and Dentistry, Delegatskaya St., 20, p. 1, Moscow 127473, Russia; 3 Microbiology Department, School of Medicine, Universidad Complutense, Avda. Complutense s/n, 28040 Madrid, Spain; 4 Instituto de Cirugía Oral Avanzada-ICOA, Calle de Fray Luis de León, 14, 28012 Madrid, Spain; 5 Instituto de Materiales, iMATUS-USC, Santiago de Compostela, Avenida do Mestre Mateo 25, 15782 La Coruña, Spain; 6 Instituto de Ciencia de Materiales de Madrid (ICMM), Consejo Superior de Investigaciones Científicas (CSIC), Campus de Cantoblanco, Calle Sor Juana Inés de la Cruz 3, 28049 Madrid, Spain

**Keywords:** zirconia, tantalum, ceramic–metal composites, implants, *Streptococcus oralis*, biofilm, bone growth

## Abstract

The surface adhesion of bacterial cells and the in vivo biocompatibility of a new ceramic–metal composite made of zirconium dioxide and tantalum were evaluated. Within the framework of an in vitro study using the crystal violet staining and colony counting methods, a relatively similar adhesion of *Streptococcus oralis* to the 3Y-TZP/Ta biocermet (roughness Ra = 0.12 ± 0.04 µm) and Ti-Al6-V4 titanium alloy (Ra = 0.04 ± 0.01 µm) was found. In addition, in an in vivo preliminary study focused on the histological analysis of a series of rods implanted in the jaws of beagle dogs for a six-month period, the absence of any fibrous tissue or inflammatory reaction at the interface between the implanted 3Y-TZP/Ta biocermets and the new bone was found. Thus, it can be concluded that the developed ceramic–metal biocomposite may be a promising new material for use in dentistry.

## 1. Introduction

The design of dental implant systems is the most active and dynamic area of research in the field of oral implantation. The evaluation of the quality of an implant is mainly concentrated on three aspects: the materials, surface properties and design. The main material for dental implants remains titanium and its alloys. They have high biocompatibility, mechanical strength, and corrosion resistance. On the other hand, ceramic materials have been used as an alternative to titanium dental materials as they offer better esthetic qualities. Many dense ceramics have been tested as promising candidates for odontology, but only a few of them have achieved human clinical applications. Yttria-stabilized tetragonal zirconia (3Y-TZP) is one of them due to its high mechanical properties as a result of the tetragonal-to-monoclinic phase transformation effect [1,2]. Nevertheless, the presence of any minor defects that may occur during the manufacture or clinical handling of ceramic products may lead to a final unpredictable fracture. In addition, it may spontaneously transform to its stable monoclinic form under in vivo conditions. This effect has become known as low-temperature hydrothermal degradation or aging [3,4]. Therefore, the development and creation of new materials that could combine the heterogeneous positive properties of ceramics and metals (biocermet) is a relevant task [5,6]. As in any multiphase material, the most significant challenge in biocermets is to attain materials with a superior performance by the subtle management of the individual properties of its components [7,8]. In this work, zirconium oxide (3Y-TZP) and tantalum were chosen as the starting materials. This metal is one of the best metallic bioinert materials due to a thin but very strong and chemically resistant tantalum pentaoxide (Ta_2_O_5_) film that self-forms on its surface. Due to its high adhesion rates, compared to traditional titanium or cobalt–chromium components, when facilitating and accelerating the process of fusion of the implant with living tissue, there is a low rejection rate of tantalum implants and the absence of inflammatory reactions [9,10,11,12,13,14,15,16,17]. Recently, we have developed a new zirconia ceramic matrix composite reinforced with 20 vol.% tantalum particles (3Y-TZP/Ta). This biocermet showed outstanding mechanical characteristics such as good flexural strength, hardness and fracture toughness [18,19,20,21]. The high values of its mechanical properties have been achieved due to the crack bridging of the elastic–plastic deformations of ductile metal particles associated with the transformation toughening mechanism in the zirconia matrix. In addition to its excellent properties under monotonic loading, this material also showed exceptional resistance to fatigue loading [22]. The 3Y-TZP/Ta composite also showed a higher wear resistance and lower friction coefficient, both related to the high toughness and the presence on the surface of an interfacial layer (autolubricating phase) of plastically deformed metal grains [23,24]. Besides their exceptional mechanical and tribological properties 3Y-TZP/Ta composites also exhibited high resistance to low-temperature degradation (LTD) due to reduction in the number of oxygen vacancies in the zirconia matrix because the presence of a solid solution of Ta_2_O_5_ [22,23]. It should also be noted that for manufacturing complex-shaped implants instead of traditional ones, modern processing methods are required, which have a number of advantages, including lower processing costs, less waste, high accuracy, versatility and a degree of automation [25,26]. Electrical discharge machining [27,28] is such a method. However, its application requires the material to have an electrical resistivity below 100–300 Ω·cm [29]. The developed 3Y-TZP/Ta biocermet also possesses an electrical conductivity suitable for electrical discharge machining and is hence appropriate for producing complex-shaped parts by electrical discharge machining to the required tolerance with reduced machining costs [30]. In addition, the developed material showed in vitro biocompatibility and the effective prevention of implant-associated infections (antibacterial properties) [31,32]. Notably, the biocompatible character of 3Y-TZP/Ta biocermets, alongside their mechanical and tribological properties, bridge the gap between functional and structural materials [33]. All of this makes them a potential material for future hard-tissue replacement.

Bacterial colonization is another factor that affects the clinical performance of dental materials, besides their conventional material properties. When an implant is exposed in the oral cavity, it provides a unique surface that can interact with native host bacteria, leading to plaque formation. The adherence of oral microorganisms and the subsequent formation of pathogenic biofilms on the surface of dental implants cause infections of the peri-implant tissues and eventually implant failure. Therefore, preventing adherence has been considered an effective strategy for preventing infectious diseases. The oral cavity is known to be characterized by a large species diversity of microorganisms that colonize dental implants as early as 30 min after insertion [34]. Most of them are commensals such as *Streptococcus oralis* [35,36]. Primary colonizers change the surface not only by their physical presence but also by exhibiting a different “surface-attached” phenotype with a distinct metabolic activity and surface properties, thus changing their environment and creating new niches for other bacteria to colonize [37]. *S. oralis* serves as an anchor for intermediate and late pathogenic colonizers [38,39], which contributes to the formation of biofilm [40]. Dental plaque as a biofilm plays a crucial role in the etiology and progression of the most common infections affecting humans, such as dental caries and periodontal diseases [41,42], as well as the likelihood of further development of endocarditis [43]. *S. oralis* can also enhance the pathogenicity of bacteria [44] and the virulence of Candida albicans [45,46,47,48]. At the same time, it has been shown that *S. oralis* can counteract bacterial pathogens and hence facilitate homeostasis [49,50].

The purpose of this study was to evaluate the in vitro adhesion of *S. oralis* to 3Y-TZP/Ta biocermet and compare it to Ti-Al6-V4 (90 wt.% titanium 6 wt.% aluminum, 4 wt.% vanadium) titanium alloy, the gold standard for endo-osseus dental implant production, and to characterize the factors associated with biofilm formation on the surfaces of the samples. Besides the in vitro microbial adhesion characterization of 3Y-TZP/Ta composites, their in vivo osteointegration performance and inflammatory response in the form of a series of rods implanted in the jaws of beagle dogs for a six-month period were evaluated using the zirconia matrix composition as a control material.

## 2. Results and Discussion

**Figure 1:**
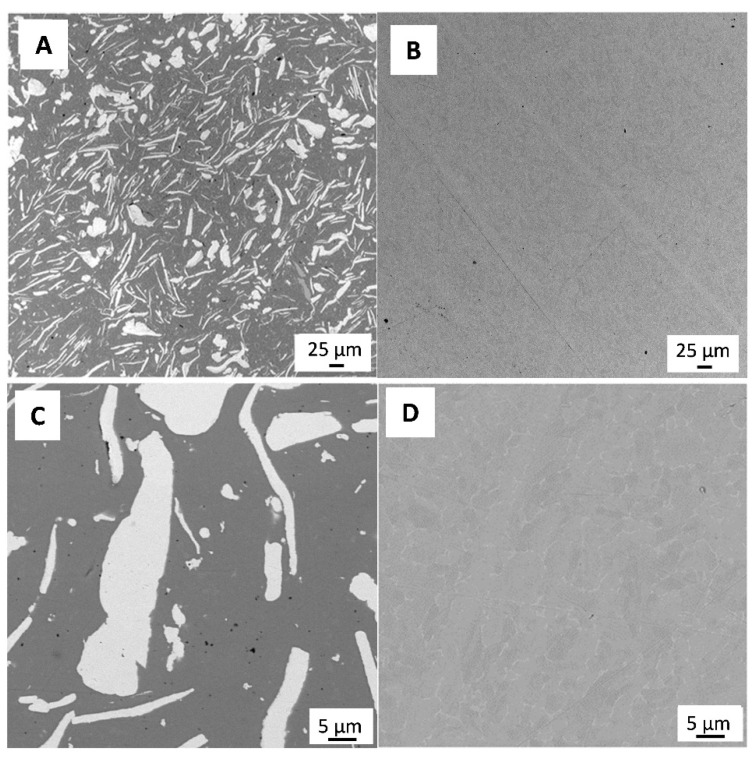
SEM image of polished surface of 3Y-TZP/Ta (**A**,**C**) and titanium alloy (**B**,**D**) disks.

Figure 1 shows a representative SEM micrograph of the polished 3Y-TZP/Ta and titanium alloy samples. The dark and grey phases in Figure 1A define 3Y-TZP and Ta grains, respectively. Tantalum particles were evenly distributed in the ceramic matrix, and no residual porosity was observed.

**Figure 2:**
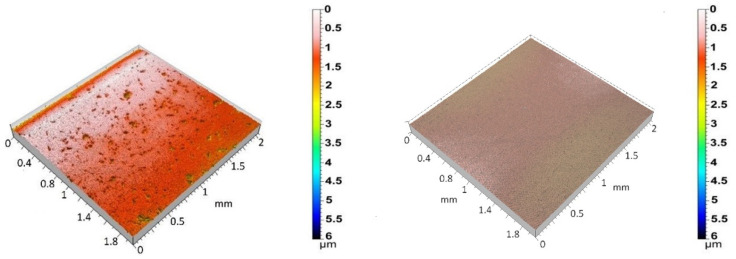
Three-dimensional surface topographies of polished 3Y-TZP/Ta (**left**) and Ti alloy (**right**) disks.

Figure 2 shows the 3D surface topographies of the 3Y-TZP/Ta biocermet and titanium disks. The 3Y-TZP/Ta biocermet disks had a much higher average Ra value (0.12 ± 0.04 µm) than the titanium disks (0.04 ± 0.01 µm). The specific surface area (roughness ratio) also followed the same pattern. It was always more than one, but it was higher for the 3Y-TZP/Ta biocermet disks (1.2 ± 0.2) than for the titanium disks (1.01 ± 0.01). These results indicate that the 3Y-TZP/Ta biocermet disks had a rougher surface and higher surface area than the titanium alloy disks.

**Figure 3:**
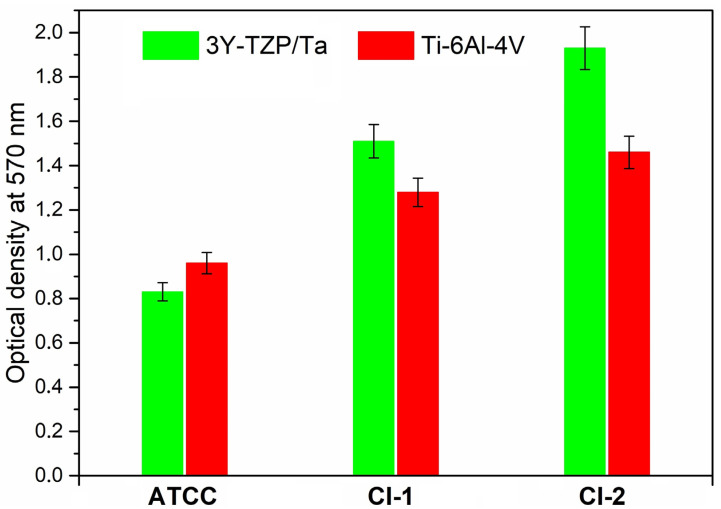
Comparison of total biofilm mass for the studied *S. oralis* strains determined by crystal violet staining among 3Y-TZP/Ta and titanium alloy disks.

Figure 3 demonstrates the total biofilm mass for all the *S. oralis* strains determined by crystal violet staining. No significant differences were observed between the absorbance values of the 3Y-TZP/Ta composite and titanium alloy disks.

**Figure 4:**
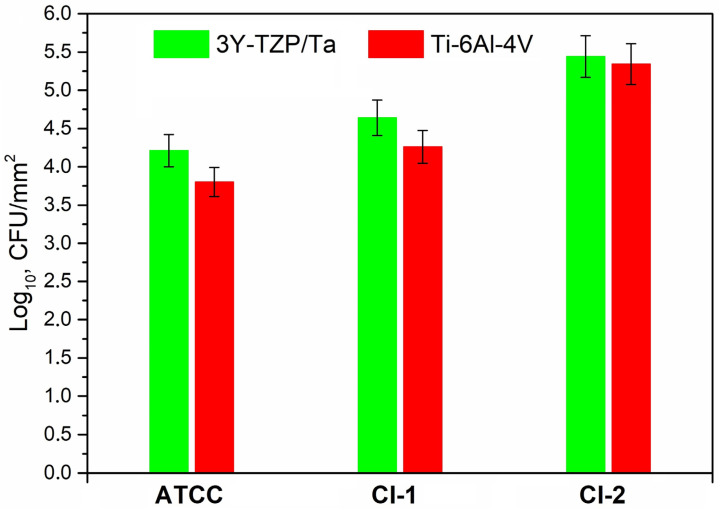
Comparison of the viable adherent bacteria (CFU/mm^2^) for the studied *S. oralis* strains among 3Y-TZP/Ta and titanium alloy disks.

Figure 4 exhibits the adherence of the viable *S. oralis* strains (log_10_ CFU/mm^2^) to the 3Y-TZP/Ta biocermet and titanium alloy surfaces. The 3Y-TZP/Ta biocermet and Ti-6Al-4V disks had no major differences in terms of the colony counts.

**Figure 5:**
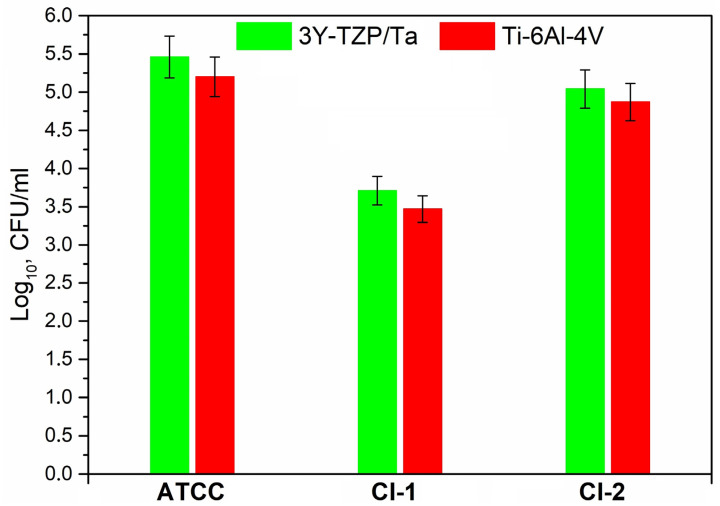
Comparison between the viable planktonic bacteria (CFU/mL) for the studied *S. oralis* strains on the surface of 3Y-TZP/Ta and titanium alloy disks.

Figure 5 shows the number of viable suspended cells (log_10_ CFU/mL) of *S. oralis* strains in THY-glucose after 24 h of incubation on the different material surfaces; no statistically significant difference was found in the number of cell colonies on the 3Y-TZP/Ta composite and titanium alloy disks.

**Figure 6:**
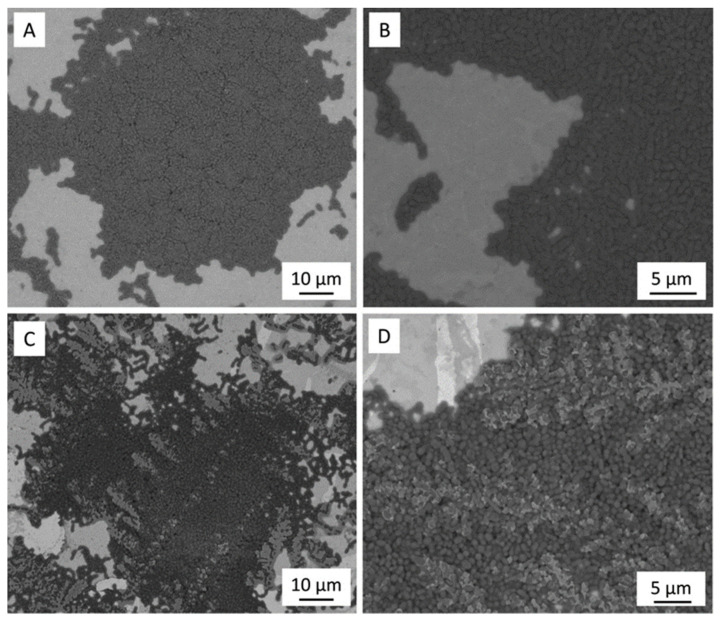
SEM micrographs after biofilm formation of the CI-1 strain on titanium alloy (**A**,**B**) and 3Y-TZP/Ta (**C**,**D**) samples.

Figure 6 shows the SEM images of the 3Y-TZP/Ta biocermet and titanium alloy disk surfaces after 24 h of incubation with CI-1. The images reveal that both materials were colonized by bacterial cells that formed dense biofilms on their surfaces.

Some material surfaces have physico-chemical properties that affect how bacteria stick to them [51]. Surface roughness is one of these properties [52,53]. Bacteria tend to stick and form biofilms more on rough surfaces than on ultra-smooth ones. This is because rough surfaces have more surface area and uneven pits that offer more sites for bacteria to grow. An Ra value of 0.2 µm is usually considered as the average roughness limit below which bacteria cannot stick [54]. Previous studies have found that smoother surfaces have less plaque formation. However, a recent study showed that titanium surfaces with hard titanium coatings like zirconium nitride or titanium nitride had fewer bacteria colonies than polished titanium, even though they had the same roughness (similar Ra values) [55]. In our study, we used two methods (crystal violet staining and colony counting) to measure the microbial amount of *S. oralis*. We found that the 3Y-TZP/Ta biocermet surface and the titanium alloy surface had similar microbial amounts, even though the biocermet surface had a higher Ra value and a larger specific surface area than the titanium alloy surface. We were not able to measure the impact of surface roughness on bacterial colonization in our study. However, some studies suggest that surface pores can shield cells from shear stress and help them stay on the surface. Based on these results, we can infer that the 3Y-TZP/Ta biocermet surfaces may have less biofilm formation and accumulation than titanium alloy surfaces with the same roughness value. This finding is not well understood yet. One possible explanation is that the biocermet surfaces have fewer oxygen defects and a more non-polar surface structure. Titanium surfaces are known to be very reactive [56,57]. They have a thin oxide layer (mainly titanium dioxide) that can adsorb both cations and anions. This layer also binds to biopolymers in saliva, creating a highly reactive surface [58]. On the other hand, zirconia implants have shown promising results in reducing biofilm formation and bacterial adhesion compared to titanium implants. A study using an anaerobic flow chamber mode tested the biofilm formation on zirconia or titanium disks with either three-species biofilm or human plaque samples [59]. The results showed that zirconia had a lower biofilm thickness and mass than titanium but a similar biofilm metabolism. This implies that zirconia implants may have less plaque accumulation and peri-implant inflammation than titanium implants. Another study measured bacterial adhesion on zirconia or titanium disks and found that the zirconia disks had lower bacterial counts than the titanium ones [60]. This finding was confirmed by other studies that also reported lower bacterial adhesion on zirconia surfaces than on pure titanium surfaces [61,62]. Furthermore, an animal model comparing zirconia or titanium implants in dogs with ligature-induced peri-implantitis showed that zirconia implants had less crestal peri-implant bone loss and no implant failure, while titanium implants had one implant loss due to peri-implantitis [63]. A recent systematic review with a meta-analysis also supported the advantage of zirconia over titanium in terms of oral biofilm parameters and surface roughness [64]. However, this effect may vary for ceramic–metal biocomposites and needs more research. Moreover, the surface free energy differences were reduced by the adsorption of salivary proteins [65], and other oral bacteria may also have different patterns of colony formation. Therefore, it is necessary to extend this research to other types of materials, like zirconia, in order to have a broader knowledge of all the materials used in dental applications. More experimental validation is necessary to apply the current findings to clinical situations.

**Figure 7:**
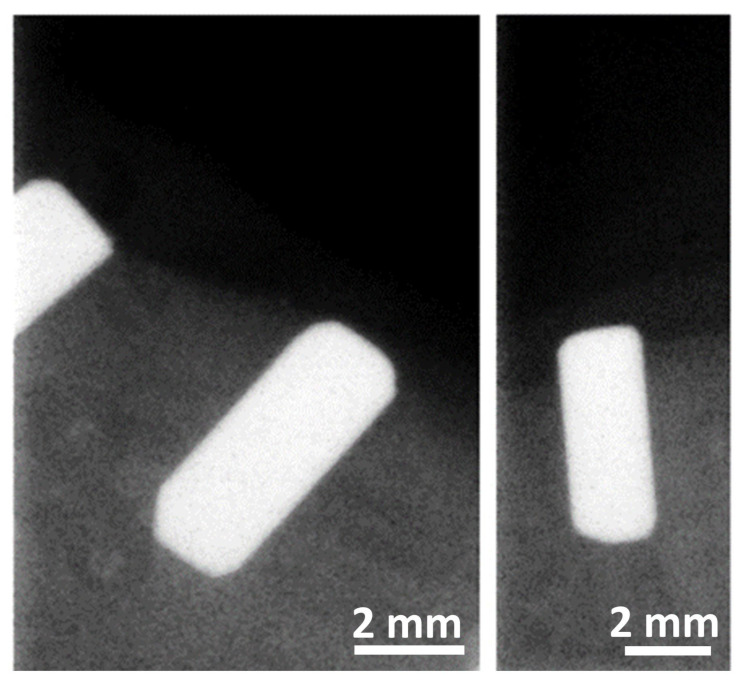
Representative digital radiographs of implanted 3Y-TZP/Ta cylinders six months after surgery.

The osteointegration performance and inflammatory response of biocermet implanted in dogs’ mandibles were also tested in an in vivo pilot study. A series of zirconia rods were also implanted as a reference group. Radiographic examination showed that the 3Y-TZP/Ta rods were well integrated with the surrounding bone six months after surgery (Figure 7). Furthermore, no visual signs of gingival inflammation, such as redness and swelling, were detected during this period.

**Figure 8:**
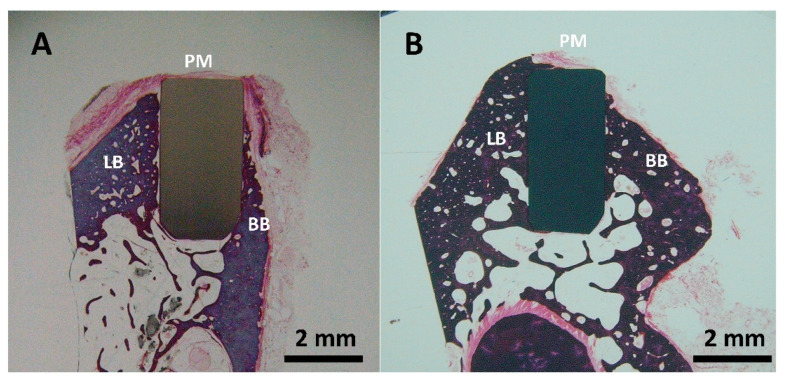
Representative buccal—lingual section of implanted ceramic (**A**) and biocermet (**B**) rods (PM: peri-implant mucosa; BB: buccal bone; LB: lingual bone).

The histological analysis of the zirconia (Figure 8A) and 3Y-TZP/Ta composite (Figure 8B) implanted rods showed good biocompatibility and osteointegration in the LB (lingual bone) and BB (buccal bone) regions after six months of implantation. The formation of new immature osteoids that directly contacted the implants without any fibrous tissue interposition was observed. No signs of inflammation, such as foreign-body giant cells or inflammatory cell infiltration, were detected in the implanted sites. It is important to point out that the amount of bone and bone-to-implant contact area appeared to be higher for the biocermet compared to the zirconia ceramic rod.

The results of the present in vivo study were largely consistent with previous investigations about the oestointegration of ZrO_2_-Nb biocermets implanted in the tibiae of New Zealand white rabbits [5]. Bartolome et al. showed that biocermets have excellent biocompatibility due to the coexistence of ceramic and metal grains and a microstructure of the composite that can act synergistically to enhance the osteointegration process. In any aqueous electrolyte, an oxide is spontaneously formed on the metal. This surface oxide is hydroxylated and has an amphoteric, or bipolar, character. The chemisorption ability of such surfaces is well known [66]. Peptides and amino acids are ligands that bind to metal oxide surfaces by replacing the hydroxyl groups. The terminal carboxyl and amino groups of amino acids and proteins bind with the surface hydroxyls. We reported in our previous study [22] that the zirconia and tantalum grains were in direct contact at the biocermet interfaces without any extra phases. A solid solution of tantalum oxide occurs, and the oxygen may be dissolved and randomly distributed in the metal. The ceramic–metal bonds create highly reactive polar oxide surfaces. Thus, ZrO_2_–Ta interfaces have a high activity to form Ta–OH groups.

In summary, this paper demonstrates that ZrO_2_–Ta biocermet has a moderate level of bacterial adhesion on its surface, is fully compatible with biological tissues, and can bond with bone. The bone formation and plaque accumulation on this ceramic/metal composite seem to be influenced by its specific microstructure, but more research is needed to elucidate the exact mechanism.

## 3. Materials and Methods

### 3.1. Materials Processing

For the fabrication of the ceramic–metal mixture, yttria-stabilized tetragonal zirconia (3Y-TZP, 3 mol% Y_2_O_3_; TZ-3YE, Tosoh Corp., Tokyo, Japan) and tantalum (99.97% purity, Alfa Aesar, Karslruhe, Germany) powders with average particle sizes of 0.26 and 44 µm, respectively, were used as the initial materials. The tantalum powder was first milled in an attritor and then wet-mixed with 80 vol% of ceramic powder. More detailed information on the starting materials and powder mixing technique are presented in previous works [20]. After homogenization, the suspension was dried for 24 h at 75 °C and then passed through a sieve with mesh size of 32 µm. The resulting mixture was consolidated using spark plasma sintering (SPS) at 1400 °C (heating rate 100 °C/min) and 80 MPa in a vacuum. The holding time at the maximum temperature was 3 min. After sintering, the oven was naturally cooled until 150 °C and then additionally supplied with argon to accelerate the process. The vacuum was broken, the chamber was door opened, and the sintered specimens were extracted. The reference samples for the in vivo studies were produced from monolithic zirconia using the same sintering cycle. The obtained samples had a diameter and thickness of 20 and 7 mm, respectively. Disks (diameter ~20 mm × 3 mm thickness) were saw-cut from a Ti-Al6-V4 (Ti017950, 99.0% purity, Good fellow, Huntingdon, England) titanium alloy rod. The titanium alloy and sintered 3Y-TZP/Ta disks then were polished with a diamond suspension from 9 to 1 μm and used in the in vitro studies. Sintered zirconia and 3Y-TZP/Ta disks were machined using a diamond tool to produce cylinders (diameter~2 mm and height~5 ± 1 mm) for the in vivo research.

### 3.2. Microstructure and Surface Characterization

The microstructure of the polished 3Y-TZP/Ta and titanium alloy disk specimens was studied using a Nova NANOSEM 230 scanning electron microscope (SEM, FEI, Hillsboro, OR, USA). A 3D surface Talysurf CLI 500 profilometer (Taylor Hobson, Leicester, UK) was used to measure the roughness ratio or specific surface area (the ratio between the actual surface and projected area, S_dr_) and the average surface roughness (Ra) of the samples by scanning the surface with a stylus. Data are presented as the mean value with the corresponding error of ten independent experiments. The stylus arm had a 90° diamond tip with a nominal radius of 2 μm. The data sampling intervals in X and Y were 0.5 and 2.5 μm, respectively. The Z-scale resolution was 32 nm. The profilometer generated a 3D map of the surface topography. The disks were rinsed with sterile saline to remove loosely attached cells after biofilm formation and then air-dried and examined by SEM.

### 3.3. In Vitro Study

#### 3.3.1. Bacterial Strains and Culture Conditions

In this study, a standard *Streptococcus oralis* ATCC 35037 and two clinical (CI-1 and CI-2) strains that were isolated from a human mouth were used [67]. Todd–Hewitt broth medium supplemented with 5% yeast extract (Difco; BD Diagnostics, Sparks, MD, USA) and 50 mM THY-glucose (Panreac, Barcelona, Spain) was used.

#### 3.3.2. Biofilm Formation Assays

To assess the biofilm formation, *S. oralis* strains were cultured in THY-glucose medium with 5% CO_2_ at 37 °C overnight on Columbia sheep blood agar plates (Difco, BD Diagnostic Systems, Sparks, MD, USA). The bacterial density was adjusted to 0.5–1 × 10^8^ CFU/mL using a UV–visible spectrophotometer (GBC, Model Cintra 101, Keysborough, Australia), and 100 µL of the bacterial suspension was added to 900 µL of the THY-glucose medium (1:10 dilution) in 24-well plates. The plates were incubated for 24 h at 37 °C in a wet chamber with 5% CO_2_. Biofilm formation was quantified by two methods: (i) crystal violet staining and absorbance measurement and (ii) viable cell counting. In order to measure the total biofilm amount, we used a crystal violet assay. We washed the disks three times with sterile saline to get rid of cells that did not stick. Then, we added 300 µL of methanol to fix the biofilm and waited for 20 min. We removed the supernatant and let the disks dry. Next, we stained the biofilm with 300 µL of 1% crystal violet solution (Química Clínica Aplicada, Tarragona, Spain) and left it for 20 min at room temperature. Finally, we washed away the extra dye with water. The biofilm amount was quantified by releasing the bound crystal violet with 200 µL of ethanol and measuring the absorbance at 570 nm using a spectrophotometer. The negative control value was subtracted from the absorbance to correct for background staining. Each experiment was repeated three times. The number of viable bacteria in the biofilm and the supernatant was determined by viable counts. The supernatant (200 µL) was collected and plated to estimate the CFU/mL of planktonic bacteria. The disks were washed three times with sterile saline to remove non-adherent cells and transferred to tubes with 5 mL of sterile saline. To release the bacteria stuck on the disk surface, the tube was shaken hard for 2 min and exposed to sound waves two times for 10 s each (Microson ultrasonic cell disruptor XL Misonix, Inc., Fanningdale, NY, USA). Then, 200 µL of the biofilm sample treated with sound waves was mixed with 0.9% saline, diluted in steps, and plated on Columbia sheep blood agar medium to count the living bacteria in CFU/mm^2^. All experiments were conducted three times using one disk per composition. The lowest number of bacteria that could be detected was 2.5 × 10^2^ CFU. Tests carried out beforehand showed that the sound waves did not kill the bacteria.

#### 3.3.3. Statistical Analysis

All statistical analyses were performed by ANOVA with the Tukey test for multiple comparisons. A *p*-value of <0.01 was considered statistically significant.

### 3.4. In Vivo Studies

Five four-year-old Beagle dogs were used. The sample size was calculated taking ethical considerations and the sample sizes used in similar studies into account. A controlled clinical trial was conducted in accordance with the ethical principles of the ARRIVE guidelines and was carried out in accordance with the UK Animals (Scientific Procedures) Act 1986, the associated guidelines, and EU Directive 2010/63/EU for animal experiments. The study protocol was approved by the Ethics Committee for Animal Research and Welfare to be carried out at the Minimally Invasive Surgery Center in Caceres (Spain). Veterinary assistance was given throughout the study. General anesthesia was achieved by intravenous injection of 10 mg/kg propofol (Propofol Hospira, Hospira Productos Farmaceuticos y Hospitalarios, Madrid, Spain). The dogs underwent endotracheal intubation with a №7 cuffed tube and were connected to a Leon Plus anesthesia machine (Heinen & Löwenstein, Bad Ems, Germany). Sevofluorane (Sevorane, Abbott Laboratories, Madrid, Spain) was used to maintain anesthesia. The dogs received ketorolac 1 mg/kg (Toradol 30 mg, Roche, Basel, Switzerland), tramadol 1.7 mg/kg (Grünenthal, Aachen, Germany), and buprenorphine 0.01 mg/kg (Buprex, Reckitt Benckiser Pharmaceuticals Limited, Berkshire, United Kingdom) for analgesia. The premolars and first molars of the lower jaw were extracted and allowed to heal for 3 months. Then, 10 cylinders per dog (3 of zirconia and 7 of biocermet) were inserted randomly in the gaps on both sides of the jaw. The dogs were fed a soft diet during this period and were euthanized by an overdose of potassium chloride (2 mEq/kg) under a premedication with dexme-detomidine (5 µg/kg) administered intravenously, followed by an overdose of propofol (15 mg/kg) administered intravenously after 6 months. Digital radiographs were obtained from all implant sections at the end of the experiment.

#### Histological Preparation and Examination

**Figure 9:**
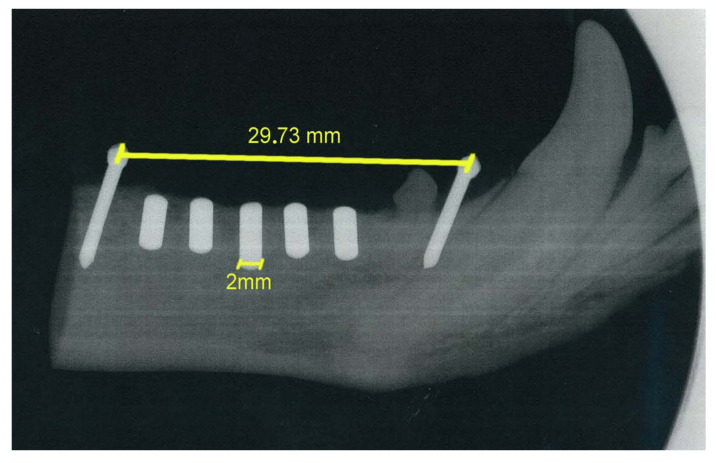
Digital radiograph of mandibular block with implanted cylinders.

The mandibular block containing the implant was removed from the mandible using an oscillating autopsy saw (Exakt, Kulzer, Germany) and stored in a 5% formaldehyde solution (pH 7). The position of the implants was confirmed by radiographic examination (Figure 9).

The specimens were immediately fixed in 4% formaldehyde and 1% calcium solution and prepared for ground sections following the protocol of Donath and Breuner [68]. Each implant block was embedded in methyl methacrylate and stained with a mixture of Harris and Wheatley Hematoxylin (Leica, Wetzlar, Germany). Two central buccal tongue grinding slides of about 25 mm were obtained from each implant. Histologic analysis was conducted using a light microscope (Optiphot, Nikon, Japan) with a digital camera (DP-12, Olympus, Japan).

## 4. Conclusions

The in vitro surface adhesion of bacterial cells and in vivo biocompatibility evaluation of a new ceramic–metal composite (biocermet) made of zirconium dioxide and tantalum were investigated. The crystal violet staining and colony counting methods showed comparable numbers of *S. oralis* microorganisms despite the higher roughness value (Ra = 0.12 μm) and larger specific surface area of the 3Y-TZP/Ta composite compared to the surface of the Ti-6Al-4V titanium alloy control sample (Ra = 0.04 μm). Based on these in vitro results, we can expect lower plaque formation on the biocermet than on the Ti-Al6-V4 surface with the same roughness values. In addition, in vivo preliminary research has shown that the surface of the 3Y-TZP/Ta metal–ceramic composite favors bone formation after 6 months in the mandible of Beagle dogs. No fibrous tissue or inflammatory reaction were observed at the interface between the 3Y-TZP/Ta implants and the new bone. This in vivo pilot study has confirmed their biocompatibility and effectiveness of osseointegration within the specified timeframe. This work revealed the clinical potential of 3Y-TZP/Ta ceramic–metal composites for dental applications. 

## Data Availability

Data are contained within the article.
